# Thermal mapping: Assessing the optimal sites for temperature measurement in the human body and emerging technologies

**DOI:** 10.14814/phy2.16155

**Published:** 2024-07-22

**Authors:** L. Yu, J. Delgado, R. De Mezerville

**Affiliations:** ^1^ Research, Development and Innovation, Establishment Labs, Coyol Alajuela Costa Rica

**Keywords:** bioheat transfer, core body temperature physiological monitoring, thermometry

## Abstract

Numerous body locations have been utilized to obtain an accurate body temperature. While some are commonly used, their accuracy, response time, invasiveness varies greatly, and determines their potential clinical and/or research use. This review discusses human body temperature locations, their accuracy, ease of use, advantages, and drawbacks. We explain the concept of core body temperature and which of the locations achieve the best correlation to this temperature. The body locations include axilla, oral cavity, rectum, digestive and urinary tracts, skin, tympanic, nasopharynx, esophagus, and pulmonary artery. The review also discusses the latest temperature technologies, heat‐flux technology and telemetric ingestible temperature pills, and the body locations used to validate these devices. Rectal and esophageal measurements are the most frequently used.

## Graphical abstract

Common temperature measurement sites and new measuremennt technologies.

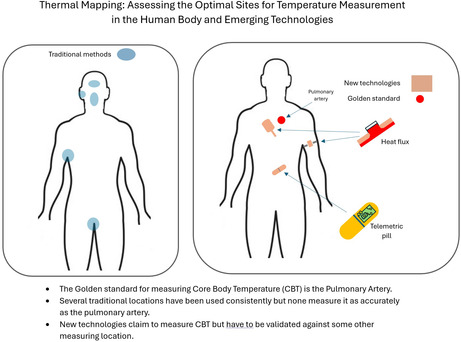

## 1 INTRODUCTION

Body temperature represents the capacity of the body to generate heat or dispose of it. It is controlled by the hypothalamus in the thermoregulator center (Loscalzo et al., 2022). In physiological terms, the body temperature is the temperature measured at a location of the human body.

Temperature is one of the four main vital signs (Neuman, 2010) (the others being heart rate, blood pressure, and breathing rate). Temperature measurement is performed on every patient, every day, in every hospital globally (Childs, 2018). Its deviation from normal ranges (between 36.5 and 37.5°C; Hymczak et al., 2021) signals the presence of an abnormal process occurring inside the body, or the effect of external conditions such as low or high ambient temperatures, high‐intensity exercise, during surgery, etc. Internal processes in which the temperature changes include cancer (Hobohm, 2001), infection (Loscalzo et al., 2022), inflammation of organs (Garami et al., 2018), cachexia (Kramer et al., 1989), or hormonal imbalances (such as hypothyroidism (Loscalzo et al., 2022)). Body temperature must be monitored continuously in certain clinical conditions, such as under general anesthesia (Sessler, 2021) (decreases in temperature), in the intensive care unit or in trauma patients.

We can measure the human body temperature in many ways, both by invasive and noninvasive means. But temperature readings are not homogeneous (Sessler, 2021) since different locations are altered by physiological and environmental issues (Hymczak et al., 2021). Thus, temperature measured in the skin and in the extremities may not reflect the actual body temperature; therefore the concept of core body temperature (CBT) was conceived (previously called deep body temperature–(Houdas & Ring, 1982)).

## 2 CORE BODY TEMPERATURE AND MEASUREMENT LOCATIONS

CBT refers to the temperature of the blood perfusing the main organs (Hymczak et al., 2021) of the body (such as the abdominal, thoracic, and cranial cavities (Lim et al., 2008; Rajbhandary & Nallathambi, 2020)), especially the thermoregulatory receptor in the hypothalamus (Chen et al., 2020). The CBT is regulated (Loscalzo et al., 2022) and maintained within a narrow range (36.5–37.5°C/97.7–99.5°F) to support normal physiologic functions (Hall, 2015). Since it is the dominant input to the autonomic system, it is the most important temperature to measure (Sessler, 2021). This temperature is the reference of internal organs to alter in case of any need: if it's lower or higher than needed. Therefore, CBT is an important sign in the diagnostic and therapeutic processes, and its reliable reading is required, especially in certain procedures such as in target temperature management. In this procedure, CBT is lowered (and closely monitored) to decrease cerebral metabolism and improve outcomes in certain conditions (brain injury, cardiac arrest) (Liu et al., 2022). Additionally, for circadian rhythm monitoring, the ideal temperature to measure is the CBT, since its nadir is used as a marker of circadian phase and is the most commonly used method (Reid, 2019).

Nonetheless, accurate CBT measurement is problematic since there are no noninvasive means of attaining it. The most accurate location for CBT measurement is the pulmonary artery (Hymczak et al., 2021; Sessler, 2021) but these measurements are very invasive in nature and not convenient for most cases.

To identify accurate alternate locations to measure temperature, we must compare these with the CBT, since some locations reflect a closer approximation than others. This paper intends to review existing literature on the most common locations to measure temperature, which ones are closer to the CBT, what devices have been developed to measure CBT and which reference temperature they are compared to.

To select the articles to review, we considered the largest academic indexes available: WoS, Scopus, Google Scholar, and Microsoft Academic. The total number of indexed papers from each index were not publicly available, but it has been reported that WoS covers over 75 million, Scopus over 76 million, Google Scholar over 300 million, and Microsoft Academic indexes over 225 million (Martín‐Martín et al., 2020). Based on this information, Google Scholar was considered the citation index that was the most comprehensive.

So, an exhaustive search was carried out in Google Scholar, with the key words: “core body temperature,” “locations,” “review.” Review articles that evaluated several body locations were selected (10 papers). The articles that were excluded were studies: performed on animals (dogs, horses, pigs, etc.) that analyzed estimation algorithms for CBT, analyzed temperature variations due to environmental conditions, or were studying CBT physiological aspects (exercise or performance‐specific studies). Articles validating CBT measurement devices were included (21 papers), since they validated the new technologies against one or more measurement locations, these include technical reports from commercial companies that were cited in these articles (2 papers: CORE Accuracy, n.d.). These two articles review several temperature location sites before describing their proprietary technologies, and several original papers evaluate them or use them as a reference.

**FIGURE 1:**
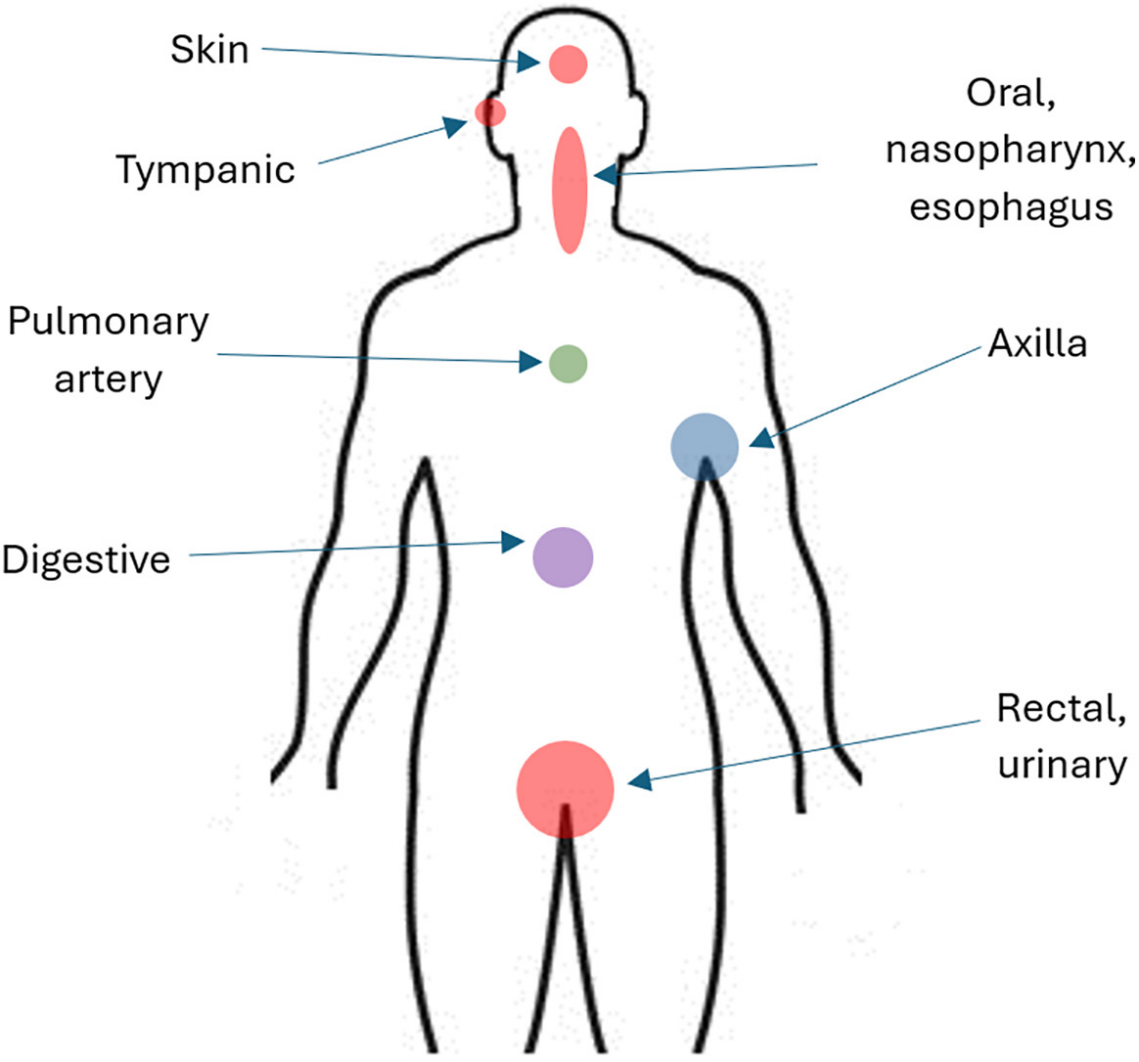
Common temperature measurement locations.

The choice of articles also reflects that in a temperature validation test, any temperature measured at a location is compared to another one. This would be independent of what temperature the body is at (e.g., normal resting temperature, after high‐intensity exercise, or in a hypothermic environment), since it is a physical variable that is compared. For the selection of articles it was not taken as crucial if the individual is resting or in exercise. The most common locations to measure temperature are shown in Figure 1 and detailed below.

### 2.1 Pulmonary artery

This is the prime location to measure CBT, but due to its invasive nature (it is measured using an artery catheter), it is not practical for most situations. It is considered the golden standard (Bergstrom, 2019; CORE Accuracy, n.d.; Hymczak et al., 2021; Liu et al., 2022; Rajbhandary & Nallathambi, 2020; Sekiguchi et al., 2019; Sessler, 2021), and its value is maintained within a narrow range, close to 37 ± 0.5°C at rest (Hymczak et al., 2021). In clinical settings, it is monitored mainly in cardiac surgery patients. There were no reviews stating that this measurement location was considered invalid.

### 2.2 Axilla

Armpit temperature measurements are not considered precise (Bergstrom, 2019; Casa et al., 2007; Edwards et al., 2002; Ganio et al., 2009; Hymczak et al., 2021; Sekiguchi et al., 2019) for several reasons: it can be affected by ambient temperature, sweating, evaporation, and dislocation. Since it is safe, accessible, and comfortable, it is commonly used (both in home and hospital settings), but it is usually lower than the CBT (Dräger Medical GmbH, n.d.; Marui et al., 2017).

### 2.3 Esophageal

The esophagus is near the left atrium and left ventricle, making it a preferred location to measure temperature. This site quickly reflects any change in temperature (Daanen et al., 2023; Imani et al., 2016; Liu et al., 2022; Sekiguchi et al., 2019; Sessler, 2021). It correlates closely with the pulmonary artery temperature, and it is used for intubated patients (Hymczak et al., 2021; Sessler, 2021) since the probe used has a low propensity to dislodge (Dräger Medical GmbH, n.d.). For other uses, it is not practical since it is very uncomfortable for patients (CORE Accuracy, n.d.) and is not as accurate as the pulmonary artery (Bergstrom, 2019).

### 2.4 Digestive tract

To access the digestive tract, telemetric pills are used. Telemetric pills are active (battery powered) integrated circuits encased in a swallowable capsule that can contain different types of sensors (see Section 3 below). They are named telemetric because the information they gather is transmitted remotely to an external reader (either wearable or not). The pills are disposable and have several days of power. These active sensors transmit temperature readings every few minutes for days (necessary for circadian rhythm studies) and have been proven to be an accurate measurement of CBT (Ganio et al., 2009; Kolka et al., 1997; Sessler, 2021), especially during exercise (Gant et al., 2006). The drawbacks include cost and the need to ingest the pill several hours before any valid readings are needed, since it must go through the pylorus to be unaffected by liquid ingestion (Casa et al., 2007; Sekiguchi et al., 2019). The pills are discarded naturally as they exit the digestive tract several days after ingestion.

### 2.5 Rectal

Rectal measurements have long been considered a very accurate measurement of the CBT (Daanen et al., 2023), have been used in research and/or sports medicine (Sekiguchi et al., 2019) extensively, especially as a reference location when evaluating other sites (Casa et al., 2007; Ganio et al., 2009). Nevertheless, even if it is a very common location to measure temperature, especially in children, some studies have shown shortcomings of using this measurement site: they have shown poor correlation with CBT (Bergstrom, 2019; Hymczak et al., 2021; Imani et al., 2016), shown delay in any change in temperature (up to 1 h depending on the contents of the rectal ampulla (Dräger Medical GmbH, n.d.; Liu et al., 2022; Sessler, 2021)) and being impractical (the probe must be inserted up to 15 cm of depth to be accurate, is uncomfortable and feces may impede the placement of the probe).

### 2.6 Skin

Skin is the outer surface of the human body, with roles that include protection from outer temperatures or dissipation of excess heat (Childs, 2018). But skin does not reflect accurately the CBT (Bergstrom, 2019; Sessler, 2021), and only reflects the skin temperature (Hymczak et al., 2021). The skin is very sensitive to external factors such as sun exposure, cold air, and internal factors such as perspiration and vasoconstriction (Casa et al., 2007; Dräger Medical GmbH, n.d.; MacRae et al., 2018; Sessler, 2021). The commonly used reference value for average skin temperature is 35°C (Mehnert et al., 2000). This is the temperature that conventional noncontact IR temperature sensors obtain while aimed at a patient's forehead.

### 2.7 Tympanic membrane

The tympanic membrane is well perfused with blood from the central sites, so some studies consider it a good location to evaluate CBT (Sessler, 2021) (it is widely measured with an infrared sensor). Other studies compare its accuracy to rectal and urinary locations (Matsukawa et al., 1993). Nonetheless, many authors refrain to consider it valid (Bergstrom, 2019; Casa et al., 2007; Ganio et al., 2009; Sekiguchi et al., 2019): it is in direct contact with the environment (no isolation from the outside temperature) (Hymczak et al., 2021), the auditory canal might be obstructed, or the measurement is wrongly made to the auditory canal instead of the membrane (Daanen et al., 2023; Dräger Medical GmbH, n.d.).

### 2.8 Nasopharynx

It is a reliable location to measure CBT since the nasal probe can be placed near the carotid arteries (Hymczak et al., 2021; Liu et al., 2022). Because it is invasive must be placed 10–20 cm past the nares (Sessler, 2021), it is used only during surgical procedures (Dräger Medical GmbH, n.d.), but it can lead to errors in measurement due to unstable circulation, imprecise probe placement, or mechanical obstruction.

### 2.9 Oral

Oral measurements are one of the most common ways to access body temperature. But foodstuffs, mucosal inflammation, or air could hamper the accuracy of its measurement (Dräger Medical GmbH, n.d.), which could be a more or less accurate approximation of CBT (CORE Accuracy, n.d.). Other studies (Sekiguchi et al., 2019) have listed several challenges to the validity of this location, including environmental factors (Ganio et al., 2009), food, smoking, and location within the mouth cavity (Bergstrom, 2019; Hymczak et al., 2021). These factors produce a lower measurement compared to CBT (Casa et al., 2007).

### 2.10 Urinary bladder

Since the urinary system receives a quarter of the cardiac output, urine should closely match the CBT (CORE Accuracy, n.d.; Hymczak et al., 2021). Urinary catheters measure the urine (in surgical procedures, or if a bladder catheter is needed), and temperature changes are reflected faster than rectal and skin locations. But in situations where the urine production is low, this measurement can be inaccurate (Bergstrom, 2019; Dräger Medical GmbH, n.d.).

### 2.11 Other sites

Some studies have suggested alternate sites, such as the skin over the carotid artery, as an accurate method to predict CBT (Imani et al., 2016; Liu et al., 2022).

### 2.12 Locations validity and golden standard

Some sites are considered valid for some authors while being invalid for others. In most cases, validity meant a useful and reliable alternative to the golden standard, the pulmonary artery. Only the pulmonary artery and the ingested pill achieved consensus that they measure CBT.

The summary of all these sites shows which are most considered valid or invalid (see Table 1).

**TABLE 1:** Temperature measurement locations. Sites are reported as valid or invalid according to the criteria of the authors of the cited papers.

Location	Valid	Not valid
Pulmonary artery	Bergstrom (2019); CORE Accuracy, (n.d.); Hymczak et al. (2021); Liu et al. (2022); Sekiguchi et al. (2019); Sessler (2021)	
Axilla		Bergstrom (2019); Casa et al. (2007); Dräger Medical GmbH (n.d.); Edwards et al. (2002); Ganio et al. (2009); Hymczak et al. (2021); Sekiguchi et al. (2019)
Esophageal	CORE Accuracy (n.d.); Daanen et al. (2023); Dräger Medical GmbH (n.d.); Hymczak et al. (2021); Imani et al. (2016); Liu et al. (2022); Sekiguchi et al. (2019); Sessler (2021)	Bergstrom (2019)
Ingested (pill)	Casa et al. (2007); Ganio et al. (2009); Gant et al. (2006); Kolka et al. (1997); Sekiguchi et al. (2019); Sessler (2021)	
Rectal	Casa et al. (2007); Daanen et al. (2023); Ganio et al. (2009); Imani et al. (2016); Sekiguchi et al. (2019)	Bergstrom (2019); Dräger Medical GmbH (n.d.); Hymczak et al. (2021); Liu et al. (2022); Sessler (2021)
Skin		Bergstrom (2019); Casa et al. (2007); Dräger Medical GmbH (n.d.); Hymczak et al. (2021); Sekiguchi et al. (2019); Sessler (2021)
Tympanic membrane	Sessler (2021)	Bergstrom (2019); Casa et al. (2007); Daanen et al. (2023); Dräger Medical GmbH (n.d.); Ganio et al. (2009); Hymczak et al. (2021); Matsukawa et al. (1993); Sekiguchi et al. (2019)
Nasopharynx	Dräger Medical GmbH (n.d.); Hymczak et al. (2021); Liu et al. (2022); Sessler (2021)	
Oral	CORE Accuracy (n.d.)	Bergstrom (2019); Casa et al. (2007); Dräger Medical GmbH (n.d.); Ganio et al. (2009); Hymczak et al. (2021); Sekiguchi et al. (2019)
Urinary bladder	Hymczak et al. (2021); CORE Accuracy (n.d.)	Bergstrom (2019); Dräger Medical GmbH (n.d.); Sessler (2021)
Skin over carotid artery	Imani et al. (2016); Liu et al. (2022)	

We must acknowledge that even if widely used locations such as skin, rectal, or nasal do not reflect CBT accurately, they have clinical value, and also have been used in comparative measurements.

## 3 VALIDATION OF NEW TECHNOLOGIES FOR CORE BODY TEMPERATURE MEASUREMENTS

As discussed in previous sections, not all locations are valid and/or convenient for the patient and the clinician, so several measuring technologies have been developed to obtain a valid measurement at a more convenient location. These technologies aim to reduce invasiveness, reduce discomfort, and aim to accurately measure (or estimate using algorithmic methods) the CBT. A thorough literature review was performed regarding new technologies that aimed to provide human CBT (described in Section 2). These devices consisted basically of two kinds of technologies: heat‐flux approach (via skin patches or chest bands) and ingestible telemetric pills.

**FIGURE 2:**
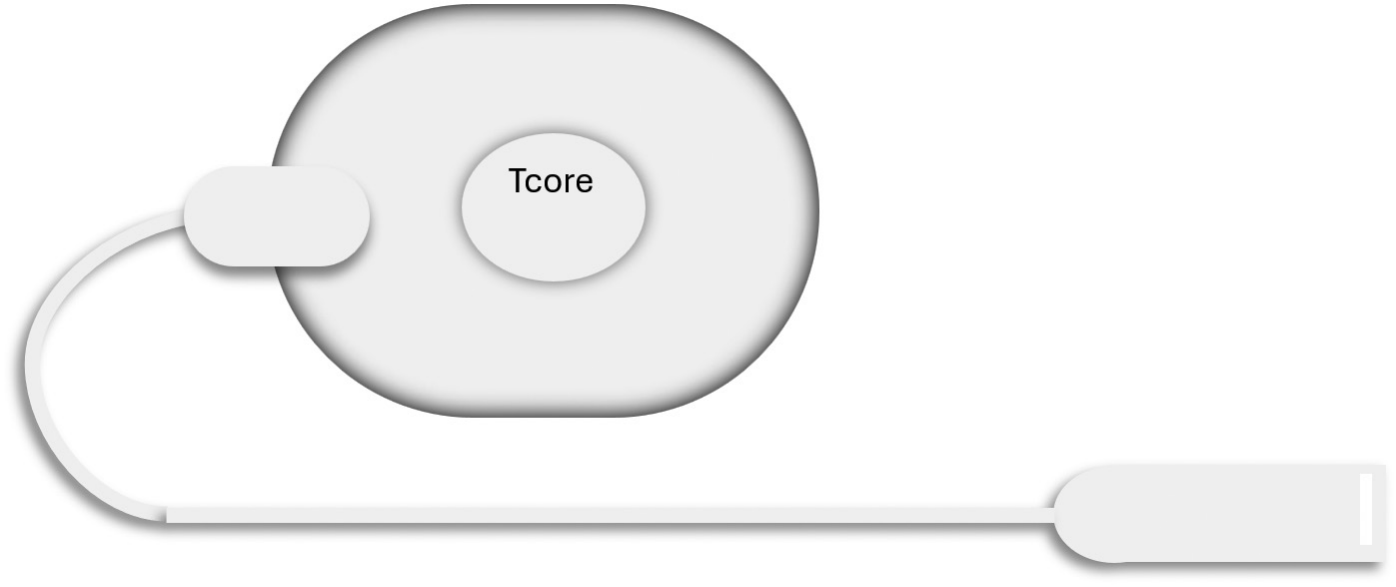
Tcore temperature sensor.

**FIGURE 3:**
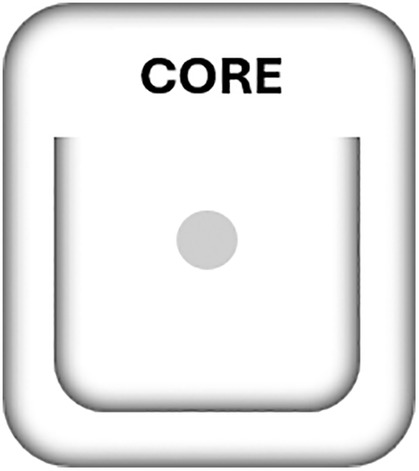
Core heat‐flux sensor.

Zero heat‐flux thermometers are thermal insulators applied to the skin, with a heater. The heater is electrically powered and is heat up to the point where there is no heat flow between the skin and the heater, at this point the temperatures are equal (Eshraghi et al., 2014). Some examples of heat‐flux thermometers are shown in Figures 2 and 3 below.

**FIGURE 4:**
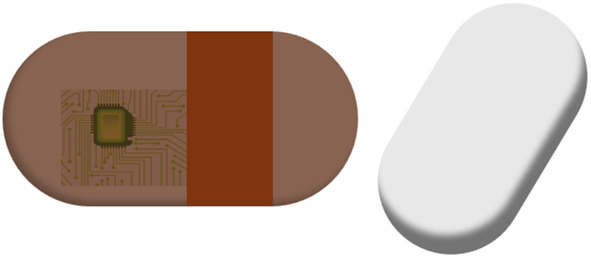
Temperature pill.

**FIGURE 5:**
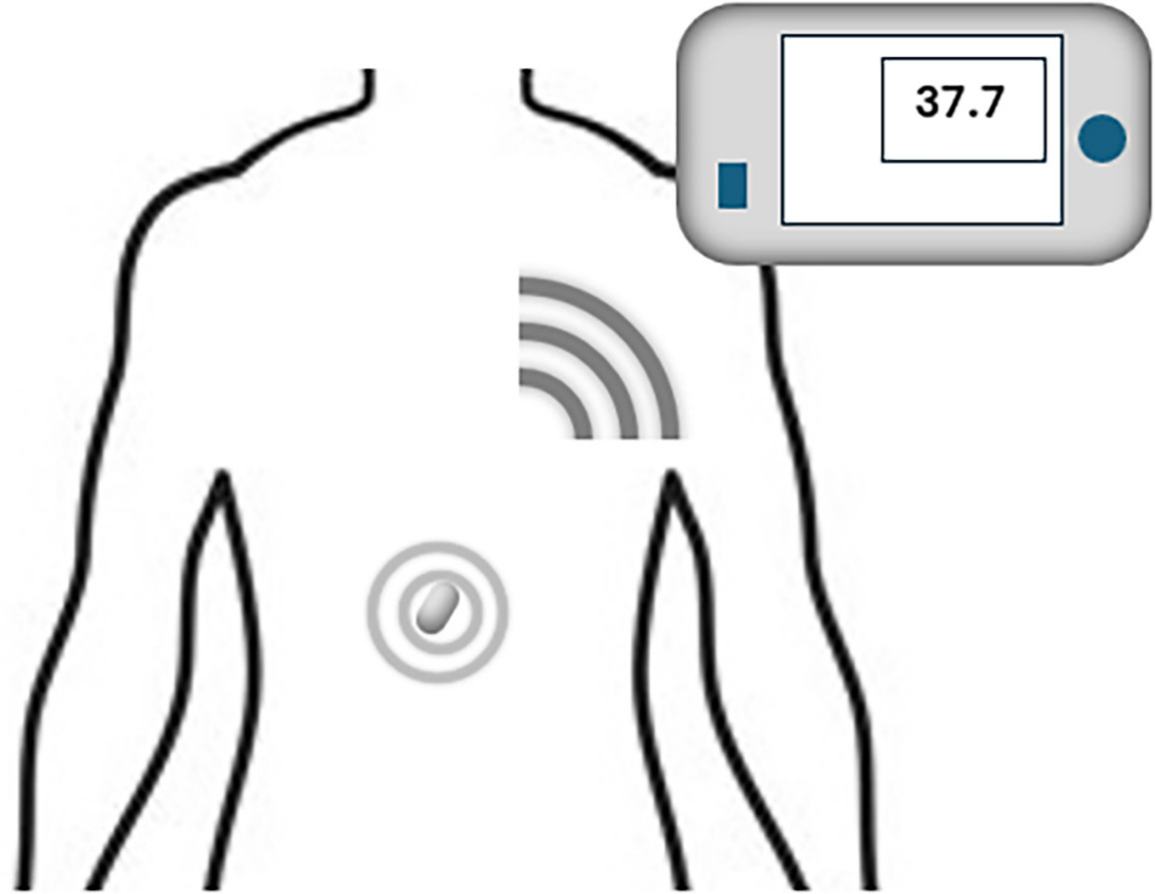
Temperature pill monitor.

Ingestible telemetric pills consist of an active battery‐powered circuit which measures temperature and transmits it to a handheld reader (McKenzie & Osgood, 2004). One instance is the BodyCap temperature pill, shown in Figure 4. The pill measures 17.7 × 8.9 mm and it is encased with a biocompatible and protective material composed of PVC (Bodycap, 2024). It usually can retain memory and power for several days, and have been used for performance evaluations (sports, military) (Byrne & Lim, 2007). It is disposed after few days naturally and cannot be reused. In this study, the ingestible sensor proved to have a high level of agreement with esophageal temperature and concluded that it represents a valid index of CBT (Figure 5).

While some authors and temperature pills suppliers recommend ingestion of the pill several hours before measurements, other authors (D'Souza et al., 2019; Notley et al., 2020) state the timing does not influence its validity. The timing of the ingestion can vary the temperature measurement (Mayer et al., 2022) both because of the location in the digestive tract, and the ingestion of liquids during measurement.

### 3.1 Selection of validation site for new measurement technologies

One of the main validation challenges of these new technologies is the selection of the measurement location for comparison. The usual locations (shown in Table 2) were esophageal, intestinal, rectal, tympanic, nasopharynx, urinary, and skin.

**TABLE 2:** Measurement technologies comparison sites.

Study	Method	Conclusion	Pulm	Axil	Eso	Int	Rect	Skin	Tymp	Naso	Oral	Urin
Janke et al. (2021)	Dräger—heat flux	Valid			x		x					
Verdel et al. (2021)	Core—heat flux	Not valid					x					
Ajčević et al. (2022)	Core—heat flux	Valid							x			
Rajbhandary and Nallathambi (2020)	VitalPatch— heat flux	Valid				x						
Goods et al. (2023)	Core—heat flux	Not valid				x						
Daanen et al. (2023)	Core—Medisim–3 M—heat flux	Not valid					x					
Byrne and Lim (2007)	Ingestible sensor	Valid			x		x					
Wagner et al. (2021)	Bair Hugger—heat flux	Valid			x		x					x
Notley et al. (2020)	Ingestible sensor	Valid					x					
Koumar et al. (2023)	Ingestible sensor	Valid			x		x					
Gosselin et al. (2019)	Ingestible sensor	Valid					x					
Easton et al. (2007)	Ingestible sensor	Valid					x		x			
Teunissen et al. (2012)	Ingestible sensor	Not valid			x		x					
O'Brien et al. (1998)	Ingestible sensor	Valid			x		x					
Bogerd et al. (2018)	Ingestible sensor	Valid					x					
Mendt et al. (2017)	Heat flux	Valid					x	x				
Mazgaoker et al. (2017)	Dräger—heat flux	Valid					x					
Zeiner et al. (2023)	Heat flux	Valid					x					
Atallah et al. (2020)	Bair Hugger–heatflux	Valid			x							
West et al. (2020)	Heat flux	Valid							x			
Morettini et al. (2020)	Bair Hugger–heat flux	Valid			x							

Even though several authors have questioned the accuracy and response time of the rectal location (see Section 2.5), most reviewed studies used it in the validation processes of the new devices. The most referenced location was rectal, followed by esophageal and intestinal.

No authors used pulmonary, axillary, nor oral locations in their publications. Some studies concluded that the readings were reliable (sufficient correlation with reference), others could not determine validity, while others were reported as valid (bias <0.1°C and 95% within ± 0.4°C), compared to their target locations. We show below in Table 2 a summary of studies and state a conclusion as “valid” if the paper stated explicitly that the location has validity or if it was considered “reliable,” otherwise we reported it as “invalid.” It was not a goal of this review to revise nor report the statistical methods used by these studies to validate their readings.

## 4 CONCLUSIONS

Body temperature varies depending on the measurement location, with the pulmonary artery being the gold standard for CBT. Nonetheless, its invasiveness makes it useful only in very specific clinical situations. For clinical trials and research activities, the ingestible pill brings enough accuracy and ease of use, while the other locations such as rectal, axillar, tympanic, do not bring sufficient accuracy to reflect real CBT.

New technologies have been developed to try to provide CBT measurements and be more convenient for the patient, but their validation studies used body locations that are not considered golden standard (pulmonary artery): mainly comparing them instead to rectal and esophageal locations. While rectal measurements are common, esophageal readings from telemetric temperature pills are more accurate but more expensive.

CBT is a vital measurement that, if measured continuously and easily, could improve diagnostic and therapeutic processes, and yield interesting research possibilities.

## AUTHOR CONTRIBUTIONS

Lochi Yu: conceptualization, methodology, writing—original draft preparation and editing, project administration. Juan José Delgado: writing—reviewing and editing. Roberto De Mezerville: writing—reviewing, supervision, funding acquisition.

## FUNDING INFORMATION

Authors are employees of Establishment Labs, which funded the work.

## CONFLICT OF INTEREST STATEMENT

All authors have reviewed and approved the final manuscript. We declare no conflicts of interest that could potentially influence the content or conclusions presented in this review.

## ETHICS STATEMENT

This manuscript is a review article and does not involve any original data collection using human subjects nor animals. Therefore, no ethics approval was required.

## Data Availability

All data presented is sourced from published academic literature. A complete list of references is provided.
